# De-Intensification from Basal-Bolus Insulin Therapy to Liraglutide in Type 2 Diabetes: Predictive Value of Mean Glycaemia during Fasting Test

**DOI:** 10.3390/life14050568

**Published:** 2024-04-28

**Authors:** Barbora Pavlikova, Martina Breburdova, Michal Krcma, Miroslav Kriz, Jan Kasparek, Zdenek Rusavy

**Affiliations:** First Department of Internal Medicine, University Hospital Pilsen, Charles University, Faculty of Medicine in Pilsen, 323 00 Pilsen, Czech Republic

**Keywords:** type 2 diabetes, therapy de-intensification, bolus-basal insulin therapy, glucagon-like peptide 1 analogues, liraglutide, fasting test

## Abstract

Background: Successful conversion from insulin therapy to glucagon-like peptide 1 receptor agonist (GLP-1RA) with basal insulin in well-controlled patients has already been demonstrated. However, the data concerning individuals with poor glycaemic control are scarce. The aim of this work was to assess the success rate of insulin therapy to liraglutide transition in poorly controlled diabetes in a real-world clinical setting and to define predictors of success. We are the first to present the method of a fasting test as a way to identify the patients at higher risk of failure after treatment de-intensification. Methods: The retrospective observational study analyzed data of 62 poorly controlled obese diabetic patients on high-dose insulin therapy, who were subjected to a 72 h fasting test during hospitalization and subsequently switched to liraglutide ± basal insulin therapy. During the fasting, all antidiabetic treatment was discontinued. Patients were classified as responders if they remained on GLP-1RA treatment after 12 months. Non-responders restarted the basal-bolus insulin (BBI) regimen. Development of glycated hemoglobin (HbA1c) and body weight in both groups, alongside with parameters associated with the higher risk of return to the BBI regimen, were analyzed. Results: A total of 71% of patients were switched successfully (=responders). Responders had more significant improvement in HbA1c (−6.4 ± 19.7 vs. −3.4 ± 22.9 mmol/mol) and weight loss (−4.6 ± 7.1 vs. −2.5 ± 4.0). Statistically significant difference between groups was found in initial HbA1c (75.6 ± 17.9 vs. 90.5 ± 23.6; *p* = 0.04), total daily dose of insulin (67.6 ± 36.4 vs. 90.8 ± 32.4; *p* = 0.02), and mean glycaemia during the fasting test (6.9 ± 1.7 vs. 8.6 ± 2.2 mmol/L; *p* < 0.01). Conclusions: This study confirms that therapy de-intensification in poorly controlled patients with a BBI regimen is possible. Higher baseline HbA1c, total daily insulin dose, and mean glucose during fasting test are negative predictive factors of successful therapy de-escalation.

## 1. Introduction

The treatment possibilities in type 2 diabetes mellitus have undergone a rapid development in the last decade. New drug groups, including drugs based on glucagon-like peptide 1 receptor agonist (GLP-1RA), were introduced to the market. Unlike sulfonylurea, the risk of hypoglycaemia is minimized as the positive effect of GLP-1RA on insulin secretion is linked to the level of glycaemia. Furthermore, GLP-1RA treatment leads to weight reduction with a further decrease in insulin resistance owing to delayed gastric emptying and a positive effect on the satiety center in the hypothalamus. In addition to the direct effect on glycaemic control, GLP-1RA treatment also has benefits resulting from its pleiotropic effect—a reduction in cardiovascular risk as well as a protective effect on pancreatic beta cells was demonstrated [1,2,3,4]. Treatment discontinuation due to gastrointestinal side effects is less frequent in long-acting substances [5].

A previously published meta-analysis demonstrated benefits of a combination of a GLP-1RA and a basal insulin over a basal-bolus insulin (BBI) regimen in patients with type 2 diabetes in the form of a (non-significant) improvement in glycated hemoglobin (HbA1c), concomitant weight reduction, and lower relative risk of hypoglycaemia [6]. Several studies demonstrated the possibility of transition from a BBI regimen to GLP-1RA therapy with or without additional basal insulin [7,8]. The most frequently proposed positive predictors of a successful switch are lower initial HbA1c, lower total daily dose of insulin, shorter diabetes duration, and various laboratory parameters indicating higher insulin secretion in the given patient (for example, higher value of fasting C-peptide or higher C-peptide level during glucagon stimulation test) [9,10,11]. However, the results of individual studies often differ due to the differences in the studied population.

In particular, there is a lack of sufficient data in patients with type 2 diabetes mellitus treated long-term with high doses of insulin or with significantly deteriorated glycaemic control (or a combination of both factors). De-intensification and simplification of therapy could improve health and consequently the quality of life of such patients. According to the American Diabetes Association^®^ and the European Association for the Study of Diabetes (ADA/EASD) guidelines, applied also in our country (Czech Republic), in the event of BBI failure, it is appropriate to consider de-escalation of therapy, ideally using GLP-1RA or sodium-glucose contransporter-2 inhibitor (SGLT2i) for their additional benefits [12]. The success rate or predictive factors of successful transition in real-life conditions in the abovementioned cohort of patients are unknown.

In the Czech Republic, the GLP-1RA therapy is reimbursed separately or in combination with basal insulin only, but not with BBI. When de-escalating from BBI, it is therefore necessary to completely discontinue meal boluses or to pay for GLP-1RA in self-pay mode. As a result, we need to choose patients for such transition carefully to prevent further deterioration of glycaemic control during the titration period of GLP-1RA.

The aim of this retrospective study was to assess the success rate of transition from BBI to GLP-1RA therapy (with or without addition of basal insulin) in a cohort of obese patients with poorly controlled type 2 diabetes, in which long-term BBI therapy had failed. We aim to derive possible predictive factors of successful therapy de-escalation. We also present a possible new marker to distinguish patients prone to GLP-1RA therapy failure—glycaemia during a 72 h long fasting test.

## 2. Materials and Methods

### 2.1. Fasting Test

Before presenting the whole study design, a thorough explanation of fasting test method is needed. In case of obese patients with poorly controlled type 2 diabetes, in which long-term BBI therapy had failed, in our clinic (First Department of Internal Medicine in University Hospital in Pilsen, Czech Republic), it is our practice to optimize the therapy and completely reeducate the patient during a planned hospitalization. Especially, if there are multiple therapy failure episodes observed during out-patient care. One of the points is the effort to de-escalate the therapy and thus enhance the probability of patient’s compliance. In patients with chronic poor glycaemic control despite intensified insulin regimen, a reduction in the resistance before the transition to GLP-1RA is attempted by three-day (72 h) fasting. In this “fasting test”, oral intake is restricted to unsweetened liquids only and all anti-diabetic medication, including insulin, is omitted. Last basal dose is administered 23 h (or more in case of insulin degludec) prior to the test and the test itself is performed only if glycaemia before start is below 10 mmol/L, normoglycaemia is preferred. During the fasting, glycaemia is frequently monitored (7 times per day), as is the presence of ketone bodies in the urine (or in the blood). If required in case of doubt, the acid-base balance parameters are monitored in order to rule out ketoacidosis in the given patient. According to the results, the test is terminated and insulin therapy resumed together with peroral intake.

At the same time, the test is used to roughly identify patients who would likely suffer from absolute insulin deficiency after a transition from BBI regimen to another form of therapy (GLP-1RA, SGLT2i). The initial pathophysiological assumption is that during starvation, the patient’s blood glucose level is affected only by gluconeogenesis in the liver. In an ideal case, this is attenuated by patient’s own insulin secretion. Glycaemia thus reflects the state between insulin resistance and partially preserved secretion of the given individual—if blood glucose is kept stable around the normal values, basal insulin secretion in the patient appears to be sufficient and treatment should be aimed at further reducing resistance and weight loss with little or no externally delivered insulin. Therefore, GLP-1RA is an appropriate option. However, if blood glucose levels continue to rise during the test, the patient’s insulin secretion may no longer be sufficient for the body’s basal needs, and conversion from BBI regimen could lead to worsening in glycaemic control and thus harm the patient.

### 2.2. Study Design

The retrospective study monitored data from 62 insulin-treated patients with type 2 diabetes mellitus, who were hospitalized in our ward for chronic inadequate glycaemic control during the period 2010–2018, had a fasting test performed in their case, and switched to liraglutide therapy with or without need of basal insulin analogue. All patients underwent a thorough education regarding diet, regimen and storage and administration of drug, including insulin. Patients switched to liraglutide treatment were instructed in the possible adverse effects of treatment, such as loss of appetite, nausea and possible vomiting, and diarrhea.

Treatment success was defined as duration of liraglutide therapy 365 days or more from initiation. Monitored patients were then divided according to the treatment success into responders (RSP) and non-responders (NRSP). Outpatient diabetologist in our facility was in charge of therapy adjustments during the monitored year. In accordance with the ADA/EASD guidelines, the dosage, possible changes or discontinuation of liraglutide treatment with back transition to BBI regimen in case of unsatisfactory glycaemic control or serious adverse effects was managed by the outpatient diabetologist. In terms of HbA1c, withdrawal of liraglutide was indicated if the improvement was smaller than 10% compared to the initial value or there was a major worsening of glycaemic control after a temporary improvement. Patients underwent outpatient visits every 2–4 months. The maximum daily dose of liraglutide was 1.2 mg per day, and the insulin dosage was not limited.

The differences in the initial parameters between the responders and non-responders were further analyzed as possible predictive factors for a successful transition. University Hospital Pilsen records were searched for the monitored parameters—gender, age, duration of diabetes, and duration of insulin therapy at the moment of hospitalization, height, body mass index (BMI), HbA1c, and total daily dose of insulin at time of admission to the hospital. One year after the index hospitalization, the development of HbA1c, body weight, total daily dose of insulin, and change in the treatment strategy (omission of liraglutide) in the patient were recorded.

**Figure 1:**
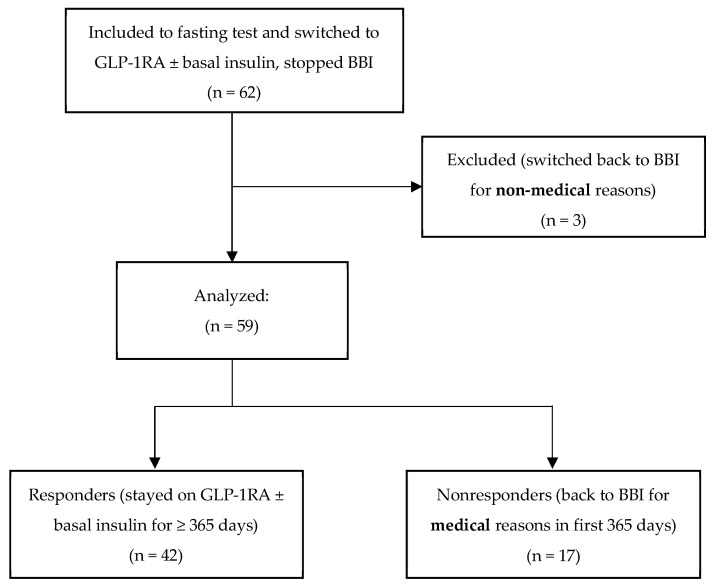
Study flow chart. GLP-1RA, glucagon-like peptide 1 agonists. BBI, basal bolus insulin regimen.

Patients with unavailable abovementioned data or where it was not possible to determine whether the discontinuation of liraglutide therapy occurred in the study period (1 year after the test) were not included in this study. Patients who discontinued therapy for non-medical reasons (drug discontinuation due to financial issues) were excluded from the analysis (Figure 1). Concomitant treatment with metformin or pioglitazone was not considered an exclusion parameter. No other concomitant antidiabetic medication was given in the studied group.

This study was conducted in compliance with the Helsinki Declaration and was approved by the Ethics Committee of the Teaching Hospital and Faculty of Medicine of Charles University in Pilsen (ref. No 105/2020). Informed consent was obtained from all subjects involved in this study.

### 2.3. Laboratory Methods

Glycaemia during the fasting test was determined by the Accu-Chek^®^ Inform II system (Roche Diagnostics), 3–7 times a day. The ketonuria during the fasting test was determined twice a day by semi-quantitative testing using diagnostic strips (Diaphan, Erba Lachema, Brno, Czech Republic). Blood glucose was measured in mmol/L (1 mmol/L = 18.0182 mg/dL). The HbA1c values were measured chromatographically (high-performance liquid chromatography using the Cobas system, Cobas 8000 Analyzer, Cobas c702 module, Roche Diagnostics, Basel, Switzerland), set in mmol/mol.

### 2.4. Statistical Methods

Statistical data analysis was performed using SAS software (version 9.4M7, SAS Institute Inc., Cary, NC, USA). Basic statistical data such as mean, standard deviation, variance, median, interquartile range, minimum, maximum were calculated for the measured parameters. For categorical variables, their absolute and relative frequencies were examined. Non-parametric tests (Wilcoxon two-sample test) were used to compare the distributions of the investigated parameters between the tested groups. Differences in frequencies were tested using Fisher’s exact test and Chi-square test. Parametric repeated ANOVA was used to evaluate the development of glycaemia over time and between groups of responders vs. non-responders. Multivariate analysis of the data was processed using logistic stepwise regression. Kaplan–Meier survival curves and the Cox regression model were used to evaluate the long-term development of treatment success. The influence of individual factors was tested using the Log-rank test, the Gehan–Wilcoxon test, and the Cox regression model. The clinical impact of individual factors in relation to treatment success was expressed using specificity, sensitivity, PV+, PV−, and Odds Ratio, or Hazard Ratio. The optimal cut-off was searched for continuous factors. Multivariate analysis was performed using the Cox regression model (stepwise regression). Statistical significance was set at alpha = 5%. Pearson’s correlation analysis was performed to analyze the correlation analysis between the examined factors with statistical significance set at alpha = 1%.

## 3. Results

The study group included 62 patients who underwent the fasting test followed by conversion to liraglutide ± basal analogue. Three patients, who then discontinued liraglutide therapy for non-medical reasons, were excluded from the group, thus 59 patients were included in the analysis. Reasons for discontinuation of therapy are listed in Table 1.

### 3.1. Basic Sample Characteritics

The entire sample was initially characterized by poor glycaemic control (mean HbA1c 80.0 ± 20.5 mmol/mol), relatively high dose of insulin (mean total daily dose 74.3 ± 36.3 IU/day), and long duration of diabetes but also of insulin therapy (14.3 and 6.4 years on average, respectively). All patients were at least overweight, and 97% were obese with the mean BMI of 35.2 ± 4.5 kg/m^2^ for the entire group. Other parameters are listed in Table 2. During the year of follow-up, 17 patients stopped liraglutide therapy (=non-responders). In 71% of patients, the GLP-1RA therapy was evaluated as successful (there was no reason to change treatment back to BBI).

### 3.2. Evaluation of Possible Predictive Factors

Initial parameters of responders and non-responders were compared to determine possible predictive factors for a successful change of therapy to liraglutide ± basal insulin (Table 2). There was a significant difference in mean glycaemia over the entire fasting test period (6.8 ± 1.6 vs. 8.6 ± 2.2 mmol/L, *p* = 0.0084), especially on day 2 of the test (6.9 ± 1.8 vs. 8.8 ± 2.5 mmol/L, *p* = 0.0064). The difference could also be observed in the patient’s initial body weight (higher in responders, 109.4 ± 13.9 vs. 97.7 ± 15.1 kg, *p* = 0.0172), although in the case of BMI, the difference was no longer statistically significant. Based on our observations, higher HbA1c could be considered as a possible negative predictive factor (75.6 ± 17.9 mmol/mol vs. 90.5 ± 23.6 mmol/mol, *p* = 0.0415). The same applies to higher total daily dose of insulin in absolute numbers or relative to the patient’s weight (67.6 ± 36.4 vs. 90.8 ± 32.4 IU/day, *p* = 0.0235, respectively, 0.62 ± 0.33 vs. 0.96 ± 0.43 IU/kg/day, *p* = 0.0057); higher values were found in non-responders.

For parameters with a possible predictive value, we evaluated the optimal cut-off, including the odds ratio for the given cut-off values (Table 3). Our study brings a new promising parameter of average glycaemia during the fasting test. If we used mean glycaemia from the whole 72 h fasting test, the optimal cut-off value was determined as 8.1 mmol/L. The odds ratio for non-response (liraglutide treatment failure in the one-year follow-up period) above this cut-off equals 5.24. Our findings also suggest that only 48 h of fasting could be enough—if the average blood glucose during the second day is higher than 8.5 mmol/L, the odds ratio of liraglutide treatment failure is 6.75. In these patients, conversion from BBI must be considered with caution, especially if other negative predictive factors are present at the same time (e.g., initial high total daily insulin dose or very poor glycaemic control).

Correlation analysis showed a strong correlation between mean glycaemias from each day of the fasting test. This suggests that it may be possible to shorten the fasting period to 48 h without losing the informative value of the test. Patients with better initial glycaemic control had lower mean glycaemias during the fasting test and better glycaemic control in the follow-up period, especially in 6 months after the transition. Lower mean glycaemias during the fasting test are positively linked with better glycaemic control after the transition. Detailed results are shown in Table 4.

### 3.3. HbA1C, Weight and Total Insulin Dose Development

In the one-year follow-up period, we observed a greater absolute decrease in the HbA1c value in responders (−6.4 ± 19.7 vs. −3.5 ± 22.9 mmol/mol, *p* = 0.588), despite a better initial glycaemic control (75.6 ± 17.7 vs. 90.1 ± 22.3 mmol/mol, *p* = 0.0415). At the same time, a greater decrease in body weight was observed when remaining on liraglutide treatment. Nonresponders were returned to BBI, but on average with a lower total daily dose of insulin (90.8 ± 31.5 vs. 79.4 ± 34.3 IU/day, *p* = 0.321). The doses of insulin almost halved with a successful switch to liraglutide treatment with complete omission of boluses. For more detailed data, see Table 5.

### 3.4. Adverse Effects

The incidence of adverse effects (AEs) of liraglutide treatment were monitored. Thirteen patients (23% of the entire sample) experienced some AEs in course of the one-year follow-up period, mostly in the first month after transition. The percentage of patients reporting AEs was similar in responders and non-responders, although non-responders tended to experience more concurrent AEs (e.g., nausea, diarrhea and abdominal pain together). Nausea (usually accompanied by vomiting), anorexia, and unspecified dyspepsia were the most frequent AEs reported by patients (Table 6).

## 4. Discussion

To our knowledge, this is one of the first studies to address the possibility of a transition from insulin therapy to GLP-1RA with or without additional basal insulin in a real-life setting and a population of poorly controlled patients with type 2 diabetes despite the treatment with high-dosed insulin. At the same time, predictive factors of a successful conversion are proposed.

The results show that a successful transition to liraglutide treatment with or without basal insulin is possible even for patients with significantly deteriorated glycaemic control (the initial mean HbA1c for responders was 75.6 ± 17.9 mmol/mol), with a long disease duration (15.0 ± 6.7 years) and a longer duration of insulin treatment (6.5 ± 5.5 years). Simultaneously, it shows that there is a population of patients in whom this transfer is not successful. According to our results, the determining factors are higher total daily dose of insulin (absolute value or related to the patient’s weight) and also higher average glycaemia during the three-day fasting test, especially during the second day of fasting.

The results of a recently published randomized study by Rosenstock et al. are comparable to our study findings [7]. Similar results when switching from BBI regimen to albiglutide ± basal insulin were documented, albeit the input HbA1c of study participants was slightly lower, the resulting improvement in compensation was slightly better, but the trends remained comparable. In contrast to our study, complete omission of boluses occurred in only 54% of patients in the albiglutide group. Therefore, the difference in HbA1c improvement may be given by the optimization of therapy by bolus insulin in case of an insufficient postprandial effect of GLP-1RA. According to the authors, it was not possible to trace predictors for a successful full conversion to GLP-1RA (i.e., complete omission of bolus insulin) in the input characteristics of patients. 

According to a prospective study by Taybani et al. [8], conversion from BBI regimen to GLP-1RA is safe in well-controlled patients (average HbA1c 46.7 ± 7.4 mmol/mol) with low daily insulin doses (43.3 ± 11.0 IU/day). Another study published on the European population by Bonora et al. [13] concluded that it was also safe in patients with a total daily dose of insulin averaging 28.6 ± 30.4 IU/day. However, for the first time in our study, patients switched from BBI regimen to GLP-1RA ± basal insulin were initially at significantly higher insulin doses and had a very poor glycaemic control. In other words, patients in whom the further therapy must be reconsidered most frequently in common clinical practice. Moreover, 71% of patients remained on the therapy after 12 months (compared with 58.6% in the Bonora et al. study [13]). This discrepancy could be caused by the fact that patients with very high glycaemias during the fasting test are not usually switched from BBI to GLP-1 RA in our center. Thus, by performing a fasting test, we make a preselection of patients that are more suitable for the switch, so the risk of failure (i.e., proportion of non-responders) is lower. 

Some similarities with our study population can be seen in the randomized study BEYOND [14], which prove the feasibility of transition from a BBI regimen to GLP-1RA + basal insulin. In the case of our patients, the glycaemic control was slightly worse (69.4 vs. 75.5 mmol/L for RSP), and the initial insulin dose was 26% higher (53.4 vs. 67.6 IU/day for RSP). Follow-up of patients in the BEYOND study lasted 6 months; therefore, the effect of a temporary improvement (due to patient motivation from the new medication) cannot be ruled out, nor can the effect of more frequent visits at the start of the transfer, as weekly phone interviews were part of the BEYOND protocol in the first months. On the contrary, our study reflects a real clinical practice, where patients are not monitored so strictly. 

Several possible predictive factors emerged from our comparison: HbA1c, total daily dose of insulin (absolute or relative to the patient’s weight), and the new parameter of average glycaemia during the fasting test.

This is the first report to present a new indicator for differentiation between the population of patients that would benefit from switching to a GLP-1RA and patients at risk of further deterioration of glycaemic control when omitting prandial insulin—average glycaemia during 72 h fasting. From the results, it seems that there is no need to maintain a three-day fasting to obtain relevant information, as the average values from the first and especially the second day of fasting were statistically significant. However, it cannot be ruled out that the third day of fasting has a positive effect on the breaking of insulin resistance and thus yields better results after the transfer. A shortened version of the test would need to be verified in a separate study. The lower the average glycaemia during fasting, the lower the risk of deterioration of glycaemic control after de-intensification of the treatment. This undemanding method can be combined with complex re-education of the patient and setting of the treatment during hospitalization.

Several Japanese studies have concluded that initial glycaemic control affects the outcome of conversion from insulin to GLP1-RA [15,16]. The possible explanation lies in the higher degree of the glucotoxic effect of hyperglycaemia in worse-controlled patients [17]. Attenuation of beta cell function then reduces the effect of GLP-1RA. Long-term noncompliance of these patients leading to deterioration in both treatment modalities could also have a similar effect. Compared to earlier studies, the average value of HbA1c in our group of responders was higher (75.6 ± 17.9 mmol/mol). Therefore, de-intensification of treatment must be considered even in such patients, ideally with simultaneous evaluation of other factors (total daily dose of insulin, glycaemia during fasting, patient compliance).

A higher total daily dose of insulin was an unfavorable prognostic factor in our group of patients. Similar results have been demonstrated in other studies dealing with a similar change in therapy in a Caucasian population [9,14,18]. Our findings (total daily dose of insulin before transfer 67.6 ± 36.4 UI/day in RSP vs. 90.8 ± 32.4 in NRSP to treatment) confirm that the total daily dose of insulin plays a role in the success of the transition. However, the exact cut-off cannot be determined from the available data, and a relatively high total daily dose of insulin should not stop the effort to simplify the treatment.

Although a higher body weight represents a positive predictive factor in our group, in case of BMI, the difference was not statistically significant. Although the result might have been affected by the small sample size, our results in this area confirm previously published meta-analysis findings [18].

Longer duration of diabetes or duration of insulin therapy are not obstacles to a successful switch. On the contrary, non-significantly higher values of these parameters in the responders’ group were observed. This contradicts some previously published results [13,16,19]. However, randomized studies in this area support our observations [7,11]. It was possible to successfully switch the therapy in patients with diabetes lasting more than 15 years and with duration of insulin therapy more than 6 years. These parameters do not have a demonstrable effect on the success of the transition from BBI regimen to GLP-1RA therapy. Therefore, their clinical use for candidate selection for the transition cannot be recommended.

The limitation of this study is its retrospective character, monocentricity, and a small group of patients, which is only representative of a certain population (Caucasian, living in Central Europe). Patients were followed in one center, however, by several physicians, which could have influenced treatment outcomes, even though these physicians follow the same guidelines. A control group of patients, who switched to GLP-1RA ± basal insulin without performing the fasting test, would also be appropriate for further comparison. 

Nevertheless, we see strengths of our study in its real-life setting and specific study population. Because of the real-life conditions, our findings can be easily applicable in everyday practice. The studied population (obese patients with type 2 diabetes and poor glycaemic control despite high doses of insulin) is the one that needs the most careful approach. They carry a higher risk of further glycaemic control deterioration and with this, a higher risk of complication of diabetes. Many failures in the history of their treatment may affect their future compliance. Therefore, every step in treatment management should be supported by evidence-based medicine. This study not only supports some findings from previous research but also expands the field to patients with worse initial glycaemic control and higher total daily insulin doses. We bring a new predictive factor of a successful switch from the BBI regimen to GLP-1RA, mean glycaemia during the fasting test. In spite of the fact that the influence of only one GLP-1RA (liraglutide) was evaluated in our study, in view of the class effect of the entire drug group, we assume similar results for other long-acting GLP-1RA.

## 5. Conclusions

Successful conversion from insulin to GLP-1RA ± basal insulin is possible even in patients with significantly poor glycaemic control or high insulin doses. At the same time, there exists a population of patient responders for whom this transfer is advantageous, both in terms of improved compensation and weight loss. Responders featured lower initial HbA1c and a lower total daily dose of insulin. In case of uncertainty in long-term poorly controlled patients with high doses of insulin, we suggest the use of prolonged fasting during hospitalization. The risk of failure of treatment de-escalation decreases with lower average fasting blood glucose. A suitable cut-off seems to be an average blood glucose value of 8.1 mmol/L during a three-day test; however, from our preliminary results, it seems that it will be possible to shorten the fasting period to 48 h without losing the informative value of the test.

## Figures and Tables

**Table 1:** Reason for discontinuation of GLP-1RA therapy (liraglutide) in absolute number of patients.

Reason for GLP-1RA Discontinuation	Number of Patients
Poor glycaemic control	15
Intolerance	2
Non-medical reasons	3

**Table 2:** Characteristics of the whole sample and comparison of initial parameters in the group of responders and non-responders.

Initial Characteristics	Entire Sample	Responders	Non-Responders	*p*
Number of patients	59	42	17	
Gender (% of women)	40	34	53	0.2537
Patients with BBI (% from entire sample)	78	81	71	0.4906
Age (years)	59.5 ± 9.5	60.2 ± 9.8	57.6 ± 9.1	0.2882
Body weight (kg)	106.0 ± 14.9	109.4 ± 13.6	97.7 ± 15.1	0.0172
BMI (kg/m^2^)	35.2 ± 4.5	35.7 ± 4.3	34.0 ± 5.2	0.1117
Duration of diabetes (years)	14.3 ± 6.2	15.0 ± 6.7	12.5 ± 4.5	0.2800
Duration of insulin therapy (years)	6.4 ± 5.0	6.5 ± 5.5	6.0 ± 3.8	0.9466
HbA1c (mmol/mol)	80.0 ± 20.5	75.6 ± 17.9	90.5 ± 23.6	0.0415
Insulin dose (IU/day)	74.3 ± 36.3	67.6 ± 36.4	90.8 ± 32.4	0.0235
Insulin dose (IU/kg/day)	0.72 ± 0.38	0.62 ± 0.33	0.96 ± 0.43	0.0057
Mean glycaemia during 72 h fasting test (mmol/L)	7.4 ± 2.0	6.9 ± 1.7	8.6 ± 2.2	0.0084
Mean glycaemia on the 1st day of fasting (mmol/L)	7.3 ± 2.0	6.8 ± 1.7	8.4 ± 2.2	0.0139
Mean glycaemia on the 2nd day of fasting (mmol/L)	7.5 ± 2.1	6.9 ± 1.8	8.8 ± 2.5	0.0064
Mean glycaemia on the 3rd day of fasting (mmol/L)	7.3 ± 2.3	6.8 ± 2.1	8.4 ± 2.5	0.0317

Data are presented as mean ± standard deviation.

**Table 3:** Determined optimal cut-off values for selected parameters and odds ratio of treatment failure for values higher than the determined optimal cut-off.

Parameter	Optimal Cut-Off Value	Odds Ratio	Confidence Interval (95%)
HbA1c (mmol/mol)	96	4.04	1.03–15.80
Insulin dose (IU/day)	77	4.32	1.28–14.62
Insulin dose (IU/kg/day)	0.87	4.78	1.41–16.27
Mean glycaemia during 72 h fasting test (mmol/L)	8.1	5.24	1.55–17.65
Mean glycaemia on the 2nd day of fasting (mmol/L)	8.5	6.75	1.87–24.42

**Table 4:** Correlation analysis between examined parameters.

	Age	BMI	DM Dur	BBI Dur	TDD	gly Total	gly d1	gly d2	gly d3	HbA1c m0	HbA1c m3	HbA1c m6	HbA1c m12
age	-	N	0.33	N	N	N	N	N	N	N	N	N	N
BMI	N	-	N	N	N	N	N	N	N	N	N	N	N
DM dur	0.33	N	-	0.49	N	N	N	N	N	N	N	N	N
BBI dur	N	N	0.49	-	0.32	N	N	N	N	N	N	N	N
TDD	N	N	N	0.32	-	0.34	0.33	0.35	N	N	0.42	N	0.43
gly total	N	N	N	N	0.34	-	0.9	0.98	0.95	0.55	0.48	0.51	N
gly d1	N	N	N	N	0.33	0.9	-	0.85	0.73	0.54	0.49	0.51	N
gly d2	N	N	N	N	0.35	0.98	0.85	-	0.93	0.55	0.47	0.54	N
gly d3	N	N	N	N	N	0.95	0.73	0.93	-	0.51	0.42	0.48	N
HbA1C m0	N	N	N	N	N	0.55	0.54	0.55	0.51	-	0.65	0.6	0.44
HbA1c m3	N	N	N	N	0.42	0.48	0.49	0.47	0.42	0.65	-	0.65	0.51
HbA1c m6	N	N	N	N	N	0.51	0.51	0.54	0.48	0.6	0.65	-	0.59
HbA1c m12	N	N	N	N	0.43	N	N	N	N	0.44	0.51	0.59	-

Pearson correlation coefficient was used for analysis, statistical significance set as *p* ≤ 0.01. N = statistically non-significant; Age = age at time of transition; DM dur = duration of diabetes; BBI dur = duration of insulin therapy; TDD = total daily dose of insulin; gly total = mean glycaemia during 72-h fasting test; gly d1 = mean glycaemia from the first day of fasting; gly d2 = mean glycaemia from the second day of fasting; gly d3 = mean glycaemia from the third day of fasting; HbA1c m0 = initial glycated hemoglobin; HbA1c m3 = glycated hemoglobin three months after switch; HbA1c m6 = glycated hemoglobin six months after switch; HbA1c m12 = glycated hemoglobin twelve months after switch.

**Table 5:** Changes in efficacy outcomes in responders and non-responders in one-year follow-up period.

Parameter	Responders			Non-Responders		
	Baseline	End	Change	Baseline	End	Change
HbA1c (mmol/mol)	75.6 ± 17.7	69.7 ± 16.0	−6.4 ± 19.7	90.1 ± 22.3 ^#^	87.3 ± 18.0 ^#^	−3.5 ± 22.9
Weight (kg)	109.4 ± 13.5	105.0 ± 14.3	−4.6 ± 7.1	97.7 ± 14.7 ^#^	94.9 ± 16.4	−2.5 ± 4.0
BMI (kg/m^2^)	35.7 ± 4.2	34.4 ± 4.4	−1.5 ± 2.2	34.0 ± 5.1	33.0 ± 6.0	−0.8 ± 1.3
Insulin dose (IU/day)	67.6 ± 36.0	34.2 ± 17.0	−33.5 ± 29.1 *	90.8 ± 31.5 ^#^	79.4 ± 34.3 ^#^	−11.4 ± 33.8 ^#^
Insulin dose (IU/kg/day)	0.62 ± 0.32	0.33 ± 0.17	−-0.29 ± 0.26 *	0.96 ± 0.41 ^#^	0.85 ± 0.38 ^#^	−0.12 ± 0.36

Data are shown as the mean ± standard deviation. * significantly different from the baseline. ^#^ significantly different from responders. *p* < 0.05 is considered to be statistically significant.

**Table 6:** Adverse effects (AEs) of liraglutide treatment reported in one-year follow-up period.

	Entire Sample	Responders	Non-Responders
Number of patients with AEs	13	9	4
% of patients with AEs	23	23	24
Nausea	5	2	3
Vomiting	4	1	3
Anorexia	4	2	2
Unspecified dyspepsia	4	4	0
Abdominal pain	2	1	1
Diarrhea	1	0	1
Other	2	2	1
Total AEs reported	22	12	11

## Data Availability

The data presented in this study are available on request from the corresponding author due to legal reasons (General Data Protection Regulation).
